# The Use of Caffeine Citrate for Respiratory Stimulation in Acquired Central Hypoventilation Syndrome: A Case Series

**DOI:** 10.2478/jccm-2023-0003

**Published:** 2023-02-08

**Authors:** Pei Ling Tan, Chuan Poh Lim, Sharon Ong, Geoffrey Sithamparapillai Samuel

**Affiliations:** 1 Singapore General Hospital, Singapore, Singapore

**Keywords:** caffeine, respiratory stimulation, acquired central hypoventilation, apnea

## Abstract

**Introductions:**

Caffeine is commonly used as a respiratory stimulant for the treatment of apnea of prematurity in neonates. However, there are no reports to date of caffeine used to improve respiratory drive in adult patients with acquired central hypoventilation syndrome (ACHS).

**Presentation of case series:**

We report two cases of ACHS who were successfully liberated from mechanical ventilation after caffeine use, without side effects. The first case was a 41-year-old ethnic Chinese male, diagnosed with high-grade astrocytoma in the right hemi-pons, intubated and admitted to the intensive care unit (ICU) in view of central hypercapnia with intermittent apneic episodes. Oral caffeine citrate (1600mg loading followed by 800mg once daily) was initiated. His ventilator support was weaned successfully after 12 days. The second case was a 65-year-old ethnic Indian female, diagnosed with posterior circulation stroke. She underwent posterior fossa decompressive craniectomy and insertion of an extra-ventricular drain. Post-operatively, she was admitted to the ICU and absence of spontaneous breath was observed for 24 hours. Oral caffeine citrate (300mg twice daily) was initiated and she regained spontaneous breath after 2 days of treatment. She was extubated and discharged from the ICU.

**Conclusion:**

Oral caffeine was an effective respiratory stimulant in the above patients with ACHS. Larger randomized controlled studies are needed to determine its efficacy in the treatment of ACHS in adult patients.

## Introductions

Acquired central hypoventilation syndrome (ACHS) occurs when the respiratory centers in the brainstem, specifically the medulla oblongata and pons, are injured [1]. It is sometimes referred to as Ondine’s Curse after a Nordic myth about a man who died in his sleep because he needed to actively control his breathing after being cursed by a water nymph [2]. ACHS poses a challenge to intensivists in weaning patients off ventilatory support, as these patients are unable to maintain an adequate minute ventilation due to a reduced respiratory rate which invariably leads to hypoxia and hypercarbia.

Caffeine has been established for the treatment and prevention of apnea of prematurity in preterm neonates [3,4]. Its stimulatory action on the respiratory centres in the brainstem and diaphragm contractility could help alleviate some of the symptoms of central hypoventilation in adults and promote the success of non-invasive ventilation strategies [5]. We describe the successful use of caffeine in 2 patients with ACHS.

## Presentation of Case Series

### Case 1

A 41-year-old ethnic Chinese male with no significant medical history was admitted to a tertiary hospital for an acute onset of left-sided weakness and numbness. He had increasingly slurred speech and unsteady gait for the past two weeks. Physical examination showed he had a left facial droop and tongue deviation to the right. He displayed left-sided weakness and dysdiadochokinesia. Magnetic resonance imaging (MRI) of brain showed an 11mm nodular-enhancing lesion in the right hemi-pons with adjacent edema, which was suspicious for neoplasm. There was no other suspicious enhancing supra-tentorial lesion. Histopathological report of a stereotactic biopsy of the right pontine lesion confirmed a high-grade astrocytoma, which was deemed by the neurosurgeon to be unsuitable for surgical intervention. Two weeks after the biopsy, the patient’s oxygen requirement increased from 40% oxygen via a facemask to 100% via non-rebreatheable mask. His respiratory rate was 35 – 40 per minute and he was using his accessory muscles of inspiration. Arterial blood gas (ABG) prior to intubation showed type 2 respiratory failure (pH 7.314 pCO_2_ 60.5 mmHg pO_2_ 84.9 mmHg SaO_2_ 96.1%). He was intubated and transferred to the neurosurgical intensive care unit (ICU) and treated for nosocomial pneumonia.

After 1 week of mechanical ventilation, he underwent tracheostomy and was ventilated with SIMV-PC (synchronized intermittent mandatory ventilation-pressure control) mode. The settings were Pi (inspiratory pressure) 12 cmH_2_O, PEEP (positive end-expiratory pressure) 5 cmH_2_O, FiO_2_ (fraction of inspired oxygen) 30%. His Glasgow Coma Scale (GCS) remained E_4_V_T_M_6_ throughout his ICU stay. To facilitate weaning from ventilatory support, the ventilatory mode was changed to pressure support ventilation. The settings were PS (pressure support) 15 cmH_2_O, PEEP 5 cmH_2_O, FiO_2_ 30%. However, SIMV-PC mode was soon resumed, due to frequent apneic episodes, bradypnea (5 – 7 breaths per minute) and hypercarbia (pH 7.398 pCO_2_ 50.0 mmHg pO_2_ 165.3 mmHg SaO_2_ 99.5%).

Given that ACHS likely caused a failure to wean from mechanical ventilation, a trial of oral caffeine was commenced for respiratory centers stimulation. An initial loading dose of caffeine citrate (1600mg) was administered via a nasogastric tube (NGT), followed by a maintenance dose of 800mg once daily. On day 2 of caffeine treatment, his respiratory rate improved (Table 1). He subsequently tolerated gradual weaning from a controlled ventilator mode to pressure support ventilation and finally to spontaneous breathing with a tracheostomy mask (FiO_2_ 28%) during the daytime, while resuming nocturnal ventilator support on SIMVPC mode. The ventilator settings were Pi 10 cmH_2_O, PS 10 cmH_2_O, PEEP 5 cmH_2_O, FiO_2_ 30%. No adverse effects of caffeine, such as tachyarrhythmia and insomnia, were observed. Due to significant improvement in his respiratory effort, he was transferred to the general ward and granted several hours of home leave, accompanied by a medical team (doctor, nurse and a respiratory therapist). He was breathing spontaneously on oxygen (5 L/min) via tracheostomy during the home visit, without any desaturation or hypoventilation and returned to the hospital uneventfully on the same day.

**Table 1:** Daily changes in mechanical ventilator setting and respiratory rate during caffeine treatment (a) Case 1

		Oral caffeine treatment												
Parameters	Baseline	Day 1	Day 2	Day 3	Day 4	Day 5	Day 6	Day 7	Day 8	Day 9	Day 10	Day 11	Day 12	Day 13
Ventilator mode	SIMV	SIMV	PSV	PSV	TM	PSV	TM	PSV	TM	SIMV	TM	TM	TM	TM
Pi	12	12	-	-	-	-	-	-	-	10	-	-	-	-
PS	12	12	12	10	-	10	-	15	-	10	-	-	-	-
PEEP	5	5	5	5	-	5	-	5	-	5	-	-	-	-
FiO2	0.3	0.3	0.3	0.3	0.28	0.3	0.28	0.3	0.28	0.3	0.28	0.28	0.28	0.28
Vent set RR	10	10	-	-	-	-	-	-	-	10	-	-	-	-
Total RR	11	11	17	19	15	15	26	10	27	13	26	28	32	28

**(b):** Case 2

		Oral caffeine treatment			
Parameters	Baseline	Day 1	Day 2	Day 3	Day 4
Ventilator mode	SIMV	SIMV	PSV	VM	VM
Pi	8	8	-	-	-
PS	8	8	8	-	-
PEEP	5	5	5	-	-
FiO_2_	0.4	0.4	0.3	0.5	0.5
Vent set RR	14	12	-	-	-
Total RR	14	12	10	15	12

FiO_2_, fraction of inspired oxygen; NRM, non-rebreathable mask; PEEP, positive end-expiratory pressure; Pi, inspiratory pressure; PS, pressure support; PSV, pressure support ventilation; RR, respiratory rate; SIMV, synchronized intermitent mandatory ventilation; TM, tracheostomy mask; VM, venturi mask

A month later, the patient had another episode of nosocomial pneumonia, necessitating full mechanical ventilation. Caffeine citrate was weaned off gradually to avoid withdrawal syndrome – 400 mg every morning for 5 days followed by 200 mg for 5 days before complete cessation. Further attempt at weaning off ventilatory support was not attempted, as the patient’s neurological status deteriorated further due to the aggressive nature of astrocytoma. He passed away two months later.

### Case 2

A 65-year-old ethnic Indian female with a medical history of diabetes mellitus, hyperlipidemia, previous left cerebellar infarct (good functional recovery), presented to the hospital with severe giddiness and vomiting. She was intubated at the emergency department for airway protection in view of her depressed GCS (E_2_V_1_M_5_). Computed tomography, including angiography of the brain revealed thrombosis in the basilar and left posterior cerebellar artery. An MRI showed acute non-hemorrhagic infarcts involving bilateral cerebellar hemispheres. There were also midbrain, left occipital lobe and hippocampus infarcts with hemorrhagic conversion. A diagnosis of posterior circulation stroke with a tip of the basilar artery thrombosis was made. The patient underwent posterior fossa decompressive craniectomy and insertion of an extra-ventricular drain within 48 hours of admission to the hospital.

Post-operatively, she was managed in the ICU on SIMV-PC mode (Pi 8 cmH_2_O, PS 8 cmH_2_O, PEEP 5 cmH_2_O, FiO_2_ 40%). Her GCS improved to E_3_V_T_M_3_ but spontaneous breaths were absent for 24 hours. She was prescribed caffeine citrate solution 300mg twice daily via a NGT on day 4 of ICU admission. After two days of caffeine therapy, she was found to be spontaneously breathing (Table 1) with GCS E_4_V_T_M_6_. She was weaned to pressure support ventilation when her respiratory rate increased (PS 8 cmH_2_O, PEEP 5 cmH_2_O, FiO_2_ 30%). Her ABG prior to extubation was pH 7.435 pCO_2_ 49.4 mmHg pO_2_ 140 mmHg SaO_2_ 99.3%. She was extubated and transferred to the general ward with 15 L/ min of oxygen via non-rebreathable mask.

As her family opted not for further escalation of care in view of a poor neurological recovery, the patient gradually declined and passed away a week later. Caffeine was continued up to the day of her demise.

## Discussion

The primary area for producing rhythmic synaptic drive for motor neurons controlling respiratory muscles is the central pattern generator located in the brainstem, specifically in the ventrolateral area of the medulla [6]. Destruction to the ventrolateral medullary region results in decreased respiratory drive. If severe, it can be fatal and explains the inability to wean from ventilator support [7]. To date, there is no standard treatment for ACHS besides supportive ICU care. This condition often results in prolonged intubation, mechanical ventilation and ICU stay as well as increased risk of nosocomial infection, leading to increased costs and healthcare resources.

Schrader et al. reported a 52-year-old male who underwent resection of left frontal grade III astrocytoma_,_ received 500 mg IV caffeine after slow emergence from anesthesia, to facilitate intraoperative language mapping [8]. In a randomized, double blind, placebo-controlled study that involved 60 patients by Gouda et al., it was found that individuals who received caffeine 500 mg had significantly faster time to extubation and fewer post-extubation respiratory complications [9]. In a retrospective study of 151 patients admitted to postanesthesia care unit (PACU), Warner et al. found that intravenous caffeine enhanced the speed of recovery following general anesthesia after a median dose of 150 mg, without adverse cardiac events including tachyarrhythmia or myocardial ischemia [10]. Fong et al. randomized 8 patients to receive IV caffeine at 7.5 mg/ kg or saline and demonstrated caffeine resulted in significantly faster emergence from isoflurane anesthesia [11].

Caffeine is commonly used in neonates for the treatment of apnea of prematurity. It is a neuro-stimulant that stimulates central respiratory drive, increasing medullary respiratory centers sensitivity to carbon dioxide and improving diaphragmatic contractility [12]. It decreases but does not eliminate apnea [13]. Caffeine also provides pain relief when used together with common analgesics [14]. It can be administered via both oral and intravenous routes. It is rapidly absorbed with minimal to no first pass metabolism. The peak plasma concentration usually occurs within 1 hour and its half-life is 5 hours in adults [12]. Therapeutic drug monitoring for caffeine is available but it is not commonly practiced in view of its wide therapeutic index. The desired therapeutic range for caffeine is 3 – 15 mg/L and the expected toxic dose is ≥ 50 mg/L [7]. Symptoms of overdose include tachycardia, restlessness, nausea and vomiting.

Caffeine citrate 20mg/mL solution was prepared by our pharmacy laboratory. Our patient in case 1 was loaded with 1600 mg of caffeine (20mg/kg body weight), maintained on 800mg once daily (10 mg/ kg body weight) while the patient in case 2 was prescribed caffeine 300mg twice daily (10 mg/kg adjusted body weight in view of BMI 35). There was no loading dose administered for the patient in case 2, as she had NGT feeds intolerance prior to caffeine therapy. With a normal gastrointestinal function and ability to tolerate feeding via NGT, the absorption of oral caffeine is likely to be complete. Assuming drug clearance follows that of the general population, the steady state maximum concentration (C_max_) and minimum concentration (C_min_) are expected to be 17.3 mg/L and 0.6 mg/L, respectively for the patient in case 1. Caffeine serum concentration was estimated to fall within therapeutic range for approximately 12 hours after each maintenance dose. Case 2 patient’s steady state C_max_ and C_min_ were expected to be 9.3 mg/L and 1.0 mg/L respectively. Caffeine serum concentration was estimated to fall within therapeutic range for approximately 16 hours a day.

In both patients, apart from the cerebral lesions, other factors causing respiratory depression such as opioids, hypothermia and metabolic conditions were absent. Cerebral inflammation and edema occur with the primary insult [15]. These may cause initial deficits that improve with time [16] due to resolution of inflammation and blood resorption in cases of hemorrhage. It is possible that the increased respiratory drive after caffeine administration could have coincided with the resolution of cerebral inflammation and edema that occurred with the primary insult. However, the time sequence of caffeine administration and ability to be weaned from mechanical ventilation suggests a possible correlation that caffeine facilitated this. These 2 cases have demonstrated the potential role of caffeine treatment in facilitating earlier liberation from mechanical ventilation, reducing ICU length of stay and cost, as well as allowing better utilization of hospital manpower and resources. Besides, it allowed the patient in case 1 to spend quality time with family during his home leave.

We chose caffeine for these 2 cases due to its availability at our institution. Besides caffeine, there are other respiratory stimulants available. These are largely divided into two main classes based on their site of action: brainstem respiratory network and carotid bodies. Table 2 summarizes the different respiratory stimulants that have been studied in human subjects [17]. These studies involved mainly surgical patients who received general anesthesia. In view of the dearth of evidence for caffeine use in the adult ICU, future studies should focus on examining its dosage regimen and efficacy in the treatment of ACHS, as well as conditions associated with depressed GCS in adult patients.

**Table 2:** Respiratory stimulants

Agent	Pharmacological class	Mechanism of action	Key findings from human study
Almitrine	Chemoreceptor stimulant	A piperazine derivative that increases the slope of respira- tory response to carbon dioxide through its long-lasting stimula- tion of ventilation [18].	Reversed fentanyl induced respiratory depression [19] Its marketing license was withdrawn in 2013 by the European Medicines Agency due to its association with potentially serious and long-lasting peripheral neuropathy [20]
Aminophylline	Phosphodiesterase (PDE) enzyme inhibitor	Inhibits PDE-3 and PDE-4 and results in bronchodilation [8]	Shortened the time to spontaneous ventilation from propofol and remifentanil anesthesia in post-operative patients [21]
Buspirone	5-HT agonist	High affinity for serotonin 5-HT_1A_ and 5-HT_2_ receptors [8]	No effect on morphine-induced respiratory depression in healthy volunteers [22]
CX717	Ampakine	Stimulates respiration through its action at AMPA receptors [23]	Prevented alfentanil induced respiratory depression without affecting analgesia, albeit an increase in sedation [24]
Doxapram	Chemoreceptor stimu- lant	stimulates respiration through its action at K_2P_ channels at type 1 glomus cells of the carotid bodies [25]	Reversed opioids induced respiratory depression as well as shortening of time to spontaneous ventilation after propofol and remifentanil anesthesia [26, 27, 28] Caused an increase in blood pressure [29] and side effects such as anxiety, panic attack and QTc prolongation [30, 31, 32]
Esketamine	NMDA receptor antago- nist	Nonselective, noncompetitive NMDA receptor antagonist [8]	Reversed alfentanil induced respiratory depression [33]
GAL021	Calcium-activated po- tassium (BK_Ca_) channel blocker	Inhibits BK_Ca_ channels primar- ily working through the carotid body [34]	Reversed alfentanil induced respiratory depression [35,36]

## Conclusion

ACHS is frequently associated with prolonged mechanical ventilation. Caffeine citrate can facilitate weaning through its neuro-stimulant effect on the respiratory centers. More randomized controlled trials are required to determine its efficacy.

## References

[1] Schläfke ME, Pokorski M, See WR, Prill RK, Loeschcke HH (1975). Chemosensitive neurons on the ventral medullary surface. Bull Physiopathol Respir (Nancy).

[2] Demartini Junior Z, Gatto LAM, Koppe GL, Francisco AN, Guerios EE (2020). Ondine’s curse: myth meets reality. Sleep Med: X 2.

[3] Clark RH, Bloom BT, Spitzer AR, Gerstmann DR (2006). Reported medication use in the neonatal intensive care unit: data from a large national data set. Pediatrics.

[4] Schmidt B, Roberts RS, Davis P (2006). Caffeine therapy for apnea of prematurity. N Engl J Med.

[5] Dobson NR, Hunt CE (2003). Pharmacology review: caffeine use in neonates: indications, pharmacokinetics, clinical effects, outcomes. NeoReviews.

[6] Bellingham MC (1998). Driving respiration: the respiratory central pattern generator. Clin Exp Pharmacol Physiol.

[7] Piper AJ, Yee BJ (2014). Hypoventilation syndromes. Compr Physiol.

[8] Schrader L, Horsfall J, Bookheimer S (2011). Use of caffeine during intraoperative awake language mapping. J Clin Neurophysiol.

[9] Gouda NM (2010). Intravenous caffeine for adult patients with obstructive sleep apnea undergoing uvulopalatopharyngoplasty: effects on postoperative respiratory complications and recovery profile. Med J Cairo Univ.

[10] Warner NS, Warner MA, Schroeder DR, Sprung J, Weingarten TN (2018). Effects of caffeine administration on sedation and respiratory parameters in patients recovering from anesthesia. Bosn J Basic Med Sci.

[11] Fong R, Wang L, Zacny JP, Khokhar S, Apfelbaum JL, Fox AP (2018). Caffeine accelerates emergence from Isoflurane anesthesia in humans: a randomized, double-blind, crossover study. Anesthesiol.

[12] (2019). Lexi-drugs online [database on the Internet].

[13] Bairam A, Uppari N, Mubayed S, Joseph V (2015). An overview on the respiratory stimulant effects of caffeine and progesterone on response to hypoxia and apnea frequency in developing rats. Adv Exp Med Biol.

[14] Derry CJ, Derry S, Moore RA (2014). Caffeine as an analgesic adjuvant for acute pain in adults. Cochrane Database Syst Rev.

[15] Lo EH, Dalkara T, Moskowitz MA (2003). Mechanisms, challenges and opportunities in stroke. Nature Reviews Neuroscience.

[16] Feeney D. M., Baron J. C. (1986). Diaschisis. Stroke.

[17] Dahan A, van der Schrier R, Smith T, Aarts L, van Velzen M, Niesters M. (2018). Averting opioid-induced respiratory depression without affecting analgesia. Anesthesiology.

[18] Stanley NN, Galloway JM, Flint KC, Campbell DB (1983). Increased respiratory chemosensitivity in patients with chronic airways obstruction. Br J Dis Chest.

[19] Gaudy JH, Dauthier C, Fourgeaux B (1982). Effect of a new analeptic drug, almitrine, on fentanyl-induced respiratory depression and analgesia in man. Br J Anaesth.

[20] (2013). European Medicines Agency Communication: Oral almitrine to be withdrawn by EU member states. EMEA/313994.

[21] Kim DW, Joo JD, In JH, Jeon YS, Jung HS, Jeon KB, Park JS, Choi JW (2013). Comparison of the recovery and respiratory effects of aminophylline and doxapram following total intravenous anesthesia with propofol and remifentanil. J Clin Anesth.

[22] Oertel BG, Schneider A, Rohrbacher M, Schmidt H, Tegeder I, Geisslinger G, Lotsch J (2007). The partial 5-hydroxytryptamine 1A receptor agonist buspirone does not antagonize morphine induced respiratory depression in humans. Clin Pharmacol Ther.

[23] Lynch G (2006). Glutamate-based therapeutic approaches: ampakines. Curr Opin Pharmacol.

[24] Oertel BG, Felden L, Tran PV, Bradshaw MH, Angst MS, Schmidt H, Johnson S, Greer JJ, Geisslinger G, Varney MA, Lötsch J (2010). Selective antagonism of opioid-induced ventilatory depression by an ampakine molecule in humans without loss of opioid analgesia. Clin Pharmacol Ther.

[25] Mitchell RA, Herbert DA (1975). Potencies of doxapram and hypoxia in stimulating carotid-body chemoreceptors and ventilation in anesthetized cats. Anesthesiology.

[26] Kim DW, Joo JD, In JH, Jeon YS, Jung HS, Jeon KB, Park JS, Choi JW (2013). Comparison of the recovery and respiratory effects of aminophylline and doxapram following total intravenous anesthesia with propofol and remifentanil. J Clin Anesth.

[27] Gairola RL, Gupta PK, Pandley K (1980). Antagonists of morphine induced respiratory depression: a study in postoperative patients. Anaesthesia.

[28] Roozekrans M, Olofsen E, van der Schrier R, Boom M, Mooren R, Dahan A (2017). Doxapram-mediated increase in cardiac output reduces opioid plasma concentrations. A PK-PD/PK-PD modeling study in healthy volunteers. Clin Pharmacol Ther.

[29] Stephen CR, Talton I (1964). Investigation of doxapram as a postanesthetic respiratory stimulant. Anesthesia & Analgesia.

[30] Abelson JL, Nesse RM, Weg JG, Curtis GC (1996a). Respiratory psychophysiology and anxiety: cognitive intervention in the doxapram model of panic. Psychosomatic Medicine.

[31] Abelson JL, Weg JG, Nesse RM, Curtis GC (1996b). Neuroendocrine responses to laboratory panic: cognitive intervention in the doxapram model. Psychoneuroendocrinology.

[32] Miyata M, Hata T, Kato N, Takeuchi M, Mizutani H, Kubota M, Yamazaki T (2007). Dynamic QT/RR relationship of cardiac conduction in premature infants treated with low-dose doxapram hydrochloride. Journal of Perinatal Medicine.

[33] Jonkman K, van Rijnsoever E, Olofsen E (2018). Esketamine counters opioid-induced respiratory depression. Br J Anaesth.

[34] McCartney CE, McClafferty H, Huibant JM, Rowan EG, Shipston MJ, Rowe IC (2005). A cysteine-rich motif confers hypoxia sensitivity to mammalian large conductance voltage- and Caactivated K (BK) channel alpha-subunits. Proc Natl Acad Sci USA.

[35] Roozekrans M, van der Schrier R, Okkerse P, Hay J, McLeod JF, Dahan A (2014). Two studies on reversal of opioid-induced respiratory depression by BK-channel blocker GAL021 in human volunteers. Anesthesiology.

[36] Roozekrans M, Olofsen E, van der Schrier R, van Gerven J, Peng S, McLeod J, Dahan A (2015). Reversal of opioid-induced respiratory depression by BK-channel blocker GAL021: A pharmacokineticpharmacodynamic modeling study in healthy volunteers. Clin Pharmacol Ther.

